# Are Urinary Tubular Injury Markers Useful in Chronic Kidney Disease? A Systematic Review and Meta Analysis

**DOI:** 10.1371/journal.pone.0167334

**Published:** 2016-12-01

**Authors:** Le-Ting Zhou, Lin-Li Lv, Ming-Ming Pan, Yu-Han Cao, Hong Liu, Ye Feng, Hai-Feng Ni, Bi-Cheng Liu

**Affiliations:** Institute of Nephrology, Zhong Da Hospital, Southeast University School of Medicine, Nanjing, Jiangsu, China; The University of Tokyo, JAPAN

## Abstract

**Background:**

Adverse outcome of chronic kidney disease, such as end stage renal disease, is a significant burden on personal health and healthcare costs. Urinary tubular injury markers, such as NGAL, KIM-1 and NAG, could provide useful prognostic value for the early identification of high-risk patients. However, discrepancies between recent large prospective studies have resulted in controversy regarding the potential clinical value of these markers. Therefore, we conducted the first meta-analysis to provide a more persuasive argument to this debate.

**Methods:**

In the current meta-analysis, based on ten prospective studies involving 29366 participants, we evaluated the role of urinary tubular injury markers (NGAL, KIM-1 and NAG) in predicting clinical outcomes including CKD stage 3, end stage renal disease and mortality. The prognostic values of these biomarkers were estimated using relative risks and 95% confidence interval in adjusted models. All risk estimates were normalized to those of 1 standard deviation increase in log-scale concentrations to minimize heterogeneity. Fixed-effects models were adopted to combine risk estimates. The quality of the research and between-study heterogeneity were evaluated. The level of research evidence was identified according to the GRADE profiler.

**Results:**

uNGAL was identified as an independent risk predictor of ESRD (pooled adjusted relative risk: 1.40[1.21 to 1.61], p<0.001) and of overall mortality (pooled adjusted relative risk: 1.10[1.03 to 1.18], p = 0.001) in patients with chronic kidney disease. A borderline significance of uKIM-1 in predicting CKD stage 3 independently in the community-based population was observed (pooled adjusted relative risk: 1.13[1.00 to 1.27], p = 0.057). Only the prognostic value of uNGAL for ESRD was supported by a grade B level of evidence.

**Conclusion:**

The concentration of uNGAL can be used in practice as an independent predictor of end stage renal disease among patients with chronic kidney disease, but it may be not useful in predicting disease progression to CKD stage 3 among community-based population.

## Introduction

Chronic kidney disease (CKD) is characterized by kidney damage or dysfunction lasting >3 months[1]. The onset of CKD stage 3 and end-stage renal disease (ESRD) are benchmarks of the progression of CKD, leading to increased mortality and health-care costs[2]. One prerequisite to improve the prognosis of CKD is to identify patients who may be at high risk of adverse outcomes early in the course of their disease. Prediction of disease progression has been explored in a wide range of studies, with several conventional risk factors, such as primary disease, level of glomerular filtration rate (GFR) and albuminuria, having been proposed[1]. Novel biomarkers are expected to yield superior performance, or at least add predictive value to conventional factors.

Urine serves as an ideal source to identify new biomarkers of kidney disease. Given that albuminuria is a marker of glomerular injury, it is reasonable to hypothesize that markers of urinary tubular injury, such as neutrophil gelatinase-associated lipocalin (NGAL), would be of clinical value in predicting disease progression in patients with CKD. Such an association between increased levels of urinary tubular injury markers and adverse CKD outcomes have been described in cross-sectional studies[3]. Several large cohorts and nested case-control studies have recently been conducted to verify the prognostic value of urinary tubular injury markers in CKD, with inconsistent conclusions obtained. As an example, although the results from the ARIC (Atherosclerosis Risk In Communities) study indicated that uNGAL is independently associated with a risk of progression to CKD stage 3[4], another large cohort from the Framingham Heart Study (FHS) provided the opposite conclusion[5]. Therefore, the goal of our systematic review was to determine whether urinary tubular injury markers are useful in predicting adverse clinical outcomes in CKD based on research evidence currently available.

## Materials and Methods

### Search strategy and selection criteria

This systematic review was performed according to the reporting guideline of the Meta-analysis of Observational Studies in Epidemiology (MOOSE)[6]. Relevant articles were identified through a search of the Web of Science, Pubmed and Cochrane Library databases, up to April 2016, and without applying a language restriction. The following search strategy was used: (chronic kidney disease OR mortality OR end-stage renal disease) AND (urine OR urinary) AND (injury marker OR neutrophil gelatinase-associated lipocalin/NGAL OR N-acetyl-b-D-glucosaminidase/NAG OR liver fatty acid-binding protein/L-FABP OR kidney injury molecule-1/KIM-1). References of relevant reviews were also searched manually to identify any eligible studies.

### Study selection

A two-stage strategy was applied for the selection of relevant studies for analysis. Titles and abstracts of each article were screened by three primary reviewers (L.T.Z, Y.H.C and Y.F), followed by a detailed full-text review to confirm eligibility. Studies were retained based on the following inclusion criteria: 1) prospective studies including cohorts, nested case-control or case-cohort studies; 2) measured tubular injury markers in urine; 3) reported incidence of CKD stage 3, ESRD and/or mortality; and 4) reported risk estimates generated by Cox or logistic model. The measurement of urinary tubular injury biomarkers was reported as a Cr-adjusted or raw concentration. As the predictive performance of Cr-adjusted and raw concentration values is similar and considering absence of a consensus regarding the benefit of adjusting levels of urinary tubular markers[7, 8], both measurements were deemed to be eligible for our analysis and their corresponding risk estimates were pooled.

Identified studies were screened according to the following exclusion criteria: 1) use of a composite end-point without separate analysis; 2) absence of adjustment for common confounding variables in analysis models; or 3) no log-transformation of urinary biomarkers. Regarding this latter exclusion criterion, reporting urinary biomarker levels on a log-scale offers better linearity with logitP and reduces heterogeneity among studies, compared with raw and categorical measures of concentration. In addition, pooling of risk estimates from two distinct scales would introduce considerable bias in our meta-analysis based on the inverse variance method. Applying this exclusion criteria to the set of identified studies resulted in the exclusion of three small-scale studies (a total of 345 participants) and were not deemed to cause a loss of the generalizability of our analysis[9–11]. Each article selected by the primary reviewers was also reviewed by an experienced supervisor (B.C.L) to confirm eligibility.

### Data extraction and quality assessment

The following information was extracted from the retained studies for analysis: study design, population, median follow-up duration, definitions of outcomes, measurement of urinary tubular injury markers, description of regression models (specifically, unit increase and adjusted covariates), and adjusted OR and HR, with their associated 95% confidence interval (CI). The quality of the research for observational studies was evaluated using the Newcastle-Ottawa Scale (NOS): http://www.ohri.ca/programs/clinical_epidemiology/oxford.asp. The NOS evaluates quality based on nine items across three categories: sample selection; comparability; and exposure/outcome.

### Statistical analysis

Risk estimates based on a 0.1 SD increase in the log-scale concentration or a ‘doubling’ in the concentration of a biomarker (i.e. 1 unit increase in log_2_X) were uniformly transformed to a 1 SD scale to minimize heterogeneity of reported outcomes across studies; the calculation procedure is shown in the supplementary materials (S1 Algorithm). HR and OR, with their 95% CI, were normalized by natural-log transformation to allow pooling of the data. As the incidence of CKD stage 3, ESRD and/or mortality in nested case-control studies was low (0.8%~2.9%), the OR reported in these studies was considered to be equivalent to RR. The Q- and I^2^-statistic were calculated to identify significant heterogeneity between studies, with a P>0.1 for the Q-statistic or I^2^<25% indicative of an acceptable level of heterogeneity for data interpretation. For studies with confirmed homogeneity, a fixed-effects model, using an inverse variance method, was adopted to combine risk estimates. Otherwise, the random-effects model was used. Potential publication bias was assessed by visual inspection of the Egger’s funnel plots and Egger’s linear regression test. All analyses were complemented using STATA (version 14.0; StataCorp LP, College Station, Texas). We also summarized our findings and provided evidence level by using GRADEprofiler (version 3.6) to support the transfer of findings to practice. A P-value <0.05 was considered statistically significant, except where specified otherwise.

## Results

### Literature search

**Fig 1:**
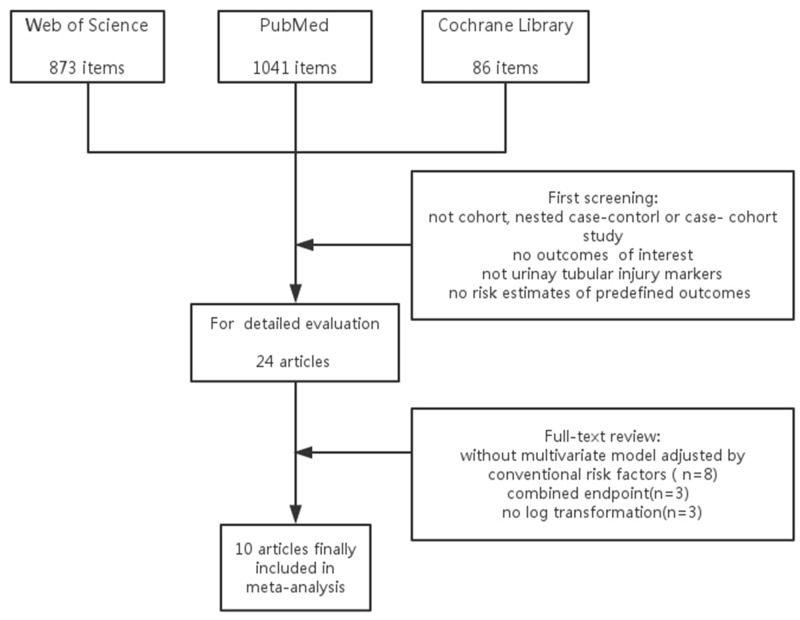
Flow Chart of Literature Search Process.

The process of literature selection is shown in Fig 1. A total of 2000 potential titles were retrieved upon initial search. After screening and further evaluation, 10 candidate articles were retained and included in our systematic analysis[4, 5, 7, 8, 12–17]. Reasons for exclusion of other retrieved titles are summarized in Fig 1.

### Study characteristics and quality

The characteristics and quality of evidence for the included studies are presented in Table 1. Seven cohort and three nested case-control studies, reporting on 29366 patients, were included in the meta-analysis. Six of these ten studies were from large research studies, including: the Chronic Renal Insufficiency Cohort (CRIC) Study, the Multi-Ethnic Study of Atherosclerosis (MESA), the ARIC Study and the FHS[4, 5, 8, 12, 14, 15]. Tubular injury markers reported upon included the NGAL, KIM-1 and NAG. Most studies had a median follow-up > 5years and reported the risk estimates based on a 1 SD increase in the log-scale of the concentration. Conventional risk factors for adverse clinical outcomes were fully-adjusted in most studies. Generally, the mean quality score of the research was 8.3/9 on the NOS scale, with all included studies meeting our quality criterion.

**Table 1:** Characteristics and quality of the 10 studies included in the meta-analysis.

First author, year ^[Ref]^	Study design	Population	Median follow-up	Outcomes	Measurement and unit increase in regression model	Adjusted covariates	NOS 1.
Fufaa 2015[13]	cohort	260 Pima Indians T2DM	14y	ESRD requiring RRT (n = 74) mortality (n = 101)	uCr-adjusted NGAL(CLIA), KIM-1(ELISA) and NAG(enzymatic assay) 1SD in log-scale	age, sex, diabetes, hypertension, HbA1c, eGFR, albuminuria	8
Peralta 2012[8]	nested case-control	202 cases and 202 controls from MESA (6,814 participants)	5y	CKD3 with eGFR decline >1 ml/min/1.73m^2^/y (incidence: 2.9%)	uCr-adjusted and unadjusted NGAL and KIM-1(ELISA) per doubling of biomarker	controls were matched for age, gender, race, diabetes and baseline eGFR models were adjusted for HTN and albuminuria	9
Bhavsar 2012[4]	nested case-control	143 cases and 143 controls from ARIC (15,792 participants)	8.6y	CKD 3 with eGFR decline> 25 (incidence: 0.9%)	unadjusted NGAL and KIM-1(ELISA) 1SD in log-scale	controls were matched for age, sex, and race models were adjusted for eGFR, SBP, antihypertensive medication use, diabetes, HDL-C, BMI, smoking albuminuria and uCr	9
Foster 2015[12]	nested case-control	135 patients with ESRD and 186 controls from ARIC	10y	ESRD defined by ICD 9 (incidence: 2.9%)	uCr-adjusted and unadjusted NGAL(CLIA), KIM-1(ELISA) and NAG (enzymatic assay) 1SD in log-scale	controls were matched for sex, race and diabetes models were adjusted for age, eGFR and albuminuria	9
Lin 2015[7]	cohort	473 advanced CKD patients of various etiologies	7y	ESRD (initiation of RRT, n = 125) mortality (n = 43)	uCr-adjusted NGAL (method not given) 1SD in log-scale	age, sex, eGFR, CVD, diabetes, HbA1c,BP, hemoglobin, albumin, CRP,BMI, cholesterol, UPCR, phosphorus	8
Liu 2013[14]	cohort	3,386 CKD patients from CRIC study	3.2 y	halving of eGFR or initiation of RRT (n = 689)	unadjusted NGAL(CLIA) 1 SD in log-scale	eGFR,24 uPr, age, sex, race, diabetes,SBP, BMI, use of ACEI or ARB, CVD, education attainment	8
Liu 2015[15]	cohort	3,386 CKD patients from CRIC study	5 y	mortality (n = 522)	uCr-adjusted and unadjusted NGAL(CLIA) 0.1 SD in log-scale	age, sex, race, eGFR, diabetes, smoking, CVD, BP, BMI, cholesterol, albuminuria, use of ARB, ACEI, aldosterone receptor antagonists, statin and antiplatelet agents	8
Seaghdha 2013[5]	cohort	2,142 participants from FHS	10.1 y	incident CKD3 without minimal eGFR decline restriction(n = 194)	uCr-adjusted NGAL and KIM-1(microsphere-based immunoassay) 1 SD in log-scale	age, sex, eGFR, BP, diabetes, dipstick proteinuria	9
Panduru 2015[17]	cohort	350 T1DM patients with macro-albuminuria	6 y	ESRD (initiation of RRT, n = 77)	uCr-adjusted KIM-1(ELISA) 1 SD in log-scale	serum triglycerides, SBP, waist to hip ratio, albuminuria	8
Mise 2016[16]	cohort	149 patients with biopsy-proven DN	2.3 y	halving of eGFR or initiation of RRT(n = 94)	uCr-adjusted NAG (enzymatic assay) 1 SD in log-scale	age, sex, BMI, diabetic retinopathy, SBP, urinary protein excretion, eGFR	7

Notes: uPCR urine protein-to-creatinine ratio; ACR: albumin-to-creatinine ratio; 24h uPr: 24h urine protein; BP: blood pressure; SBP: systolic blood pressure; BMI: body mass index; ACEI: angiotensin-converting enzyme inhibitor; ARB: angiotensin receptor blocker; ICD9: International Classification of Diseases Ninth Revision; uCr: urinary creatinine; HDL-C: high density lipoprotein-cholesterol; CRP: C-reaction protein; CVD: cardiovascular disease; CLIA: chemiluminescence immunoassay; ELISA: enzyme linked immunosorbent assay

### Predictive value of uKIM-1 and uNGAL for CKD stage 3

**Fig 2:**
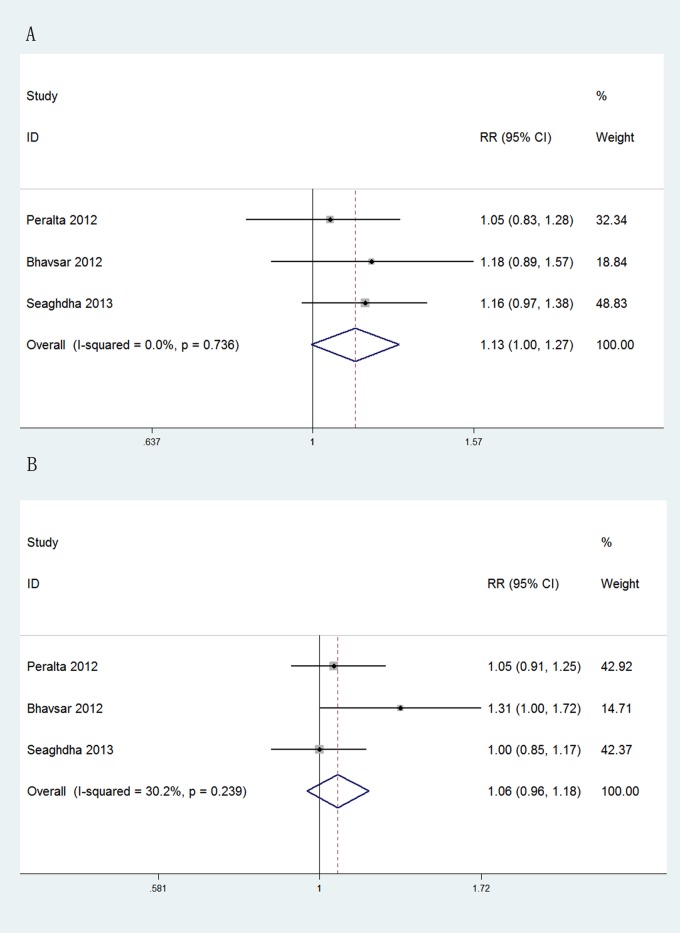
Forest plots of uKIM-1 and uNGAL in predicting CKD stage 3. (A) Pooled adjusted risk estimates for CKD stage 3 by a 1 SD increase in the log-transformed concentration of uKIM-1 in community-based population. (B) Pooled adjusted risk estimates for CKD stage 3 by a 1 SD increase in the log-transformed concentration of uNGAL in community-based population. ES: effects.

The prognostic value of uKIM-1was evaluated in three large community-based studies involving a total of 24748 participants. The risk estimates of per doubling of biomarker were transformed to those of 1SD increase in log-scale concentration[8]. As shown in Fig 2, as an independent predictive factor of CKD stage 3, uKIM-1 concentration exhibited a borderline significance (pooled adjusted RR: 1.13 [95%CI, 1.00 to 1.27], p = 0.057). In contrast, the concentration of uNGAL had lower efficacy than that of uKIM-1 concentration as a predictive factor of CKD stage 3(pooled adjusted RR: 1.06, [95%CI, 0.96 to 1.18], p = 0.253). No significant heterogeneity was observed for both analyses.

### Predictive value of uKIM-1, uNAG and uNGAL for ESRD

After pooling of the unadjusted risk estimates, all three biomarkers (uKIM-1, uNAG and uNGAL) were associated with the risk for ESRD (pooled unadjusted RR, 1.51~1.79, S1 Fig). Due to the presence of a wide range of confounding variables, all three analyses were identified as having substantial heterogeneity (I^2^, 80.8%~91.4%).

**Fig 3:**
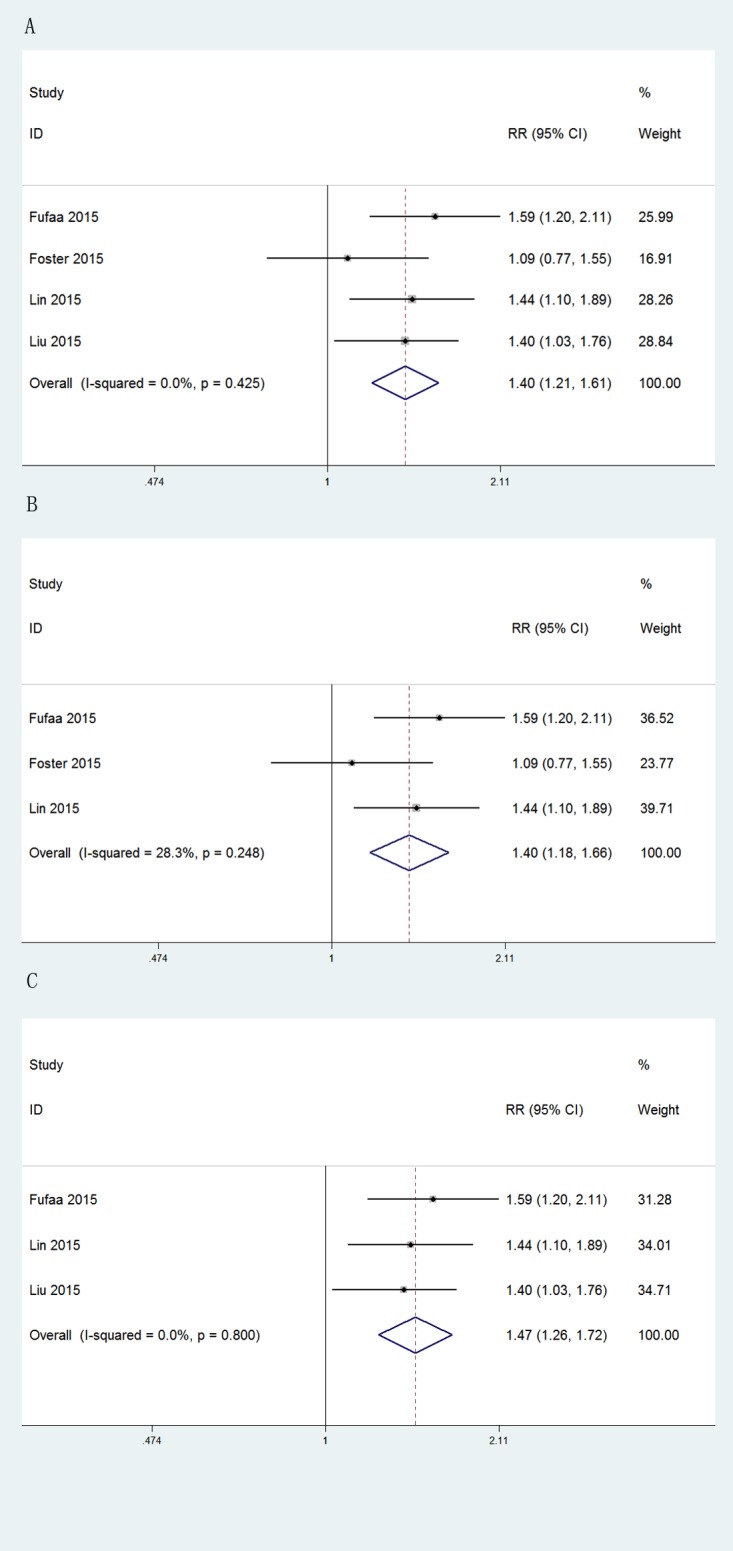
Forest plots of uNGAL in predicting ESRD. (A) Pooled adjusted risk estimates for ESRD by a 1 SD increase in the log-transformed concentration of uNGAL. (B) Subgroup analysis excluding one study in which uNGAL was reported without Cr-adjustment. (C) Subgroup analysis excluding one community-based study.

The independent prognostic value of uNGAL concentration for ESRD was further analyzed using the data of four studies including 19911 participants. As shown in Fig 3, the concentration of uNGAL was independently related to the risk of ESRD (pooled adjusted RR: 1.40, [95%CI, 1.21 to 1.61], p<0.001). Although no significant statistical heterogeneity was identified among these four studies (Q-statistic p = 0.465; I^2^ = 0%), we still conducted a subgroup analysis to further verify our results. From this subgroup analysis, we excluded the study by Liu et al.[14], which reported raw concentration values of uNGAL, and the study be Foster et al.[12], reporting data for a community-based population. After these exclusions, the calculated RR remained comparable (exclusion of Liu et al., pooled adjusted RR, 1.40, [95%CI, 1.18 to 1.66], p<0.001; exclusion of Foster et al., pooled adjusted RR, 1.47, [95%CI, 1.26 to 1.72], p<0.001). No significant heterogeneity in findings was observed.

**Fig 4:**
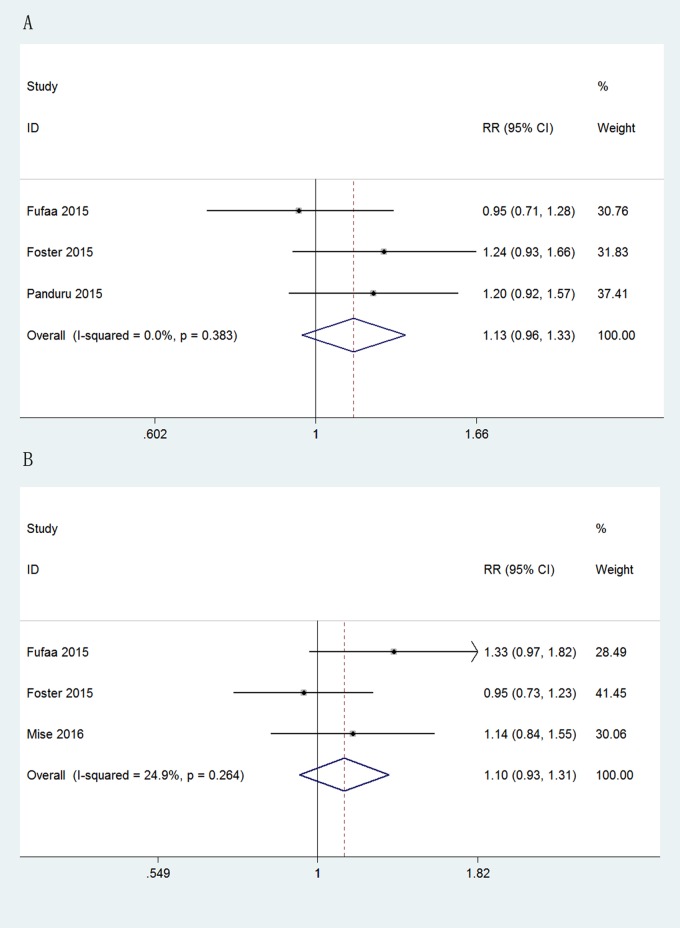
Forest plots of uKIM-1 and uNAG in predicting ESRD. (A) Pooled adjusted risk estimates for ESRD by a 1 SD increase in the log-transformed concentration of uKIM-1. (B) Pooled adjusted risk estimates for ESRD by a 1 SD increase in the log-transformed concentration of uNAG.

Evaluation of the predictive value of the concentration of uKIM-1 and uNAG for ESRD was based on the data of three studies for each marker. Fig 4 showed that neither uKIM-1 nor uNAG independently predicted the incidence of ESRD (uKIM-1, pooled adjusted RR, 1.13, [95%CI, 0.96 to 1.33], p = 0.147; uNAG, pooled adjusted RR, 1.10, [95%CI: 0.93 to 1.31], p = 0.246), with no significant heterogeneity.

### uNGAL as an independent risk factor of overall mortality in patients with CKD

**Fig 5:**
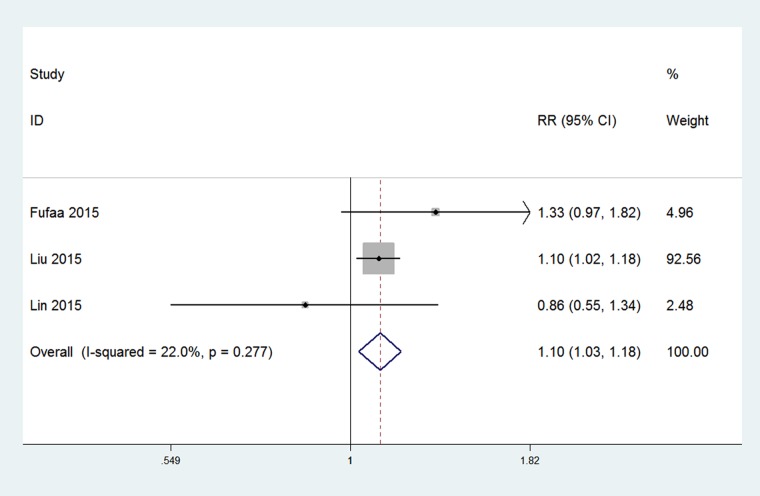
Forest plot of uNGAL in predicting mortality in patients with CKD. Pooled adjusted risk estimates for mortality by a 1 SD increase in the log-transformed concentration of uNGAL.

The overall rate of mortality in patients with CKD, from all causes, was reported in three studies involving 4119 patients with CKD. The risk estimates, based on a 0.1 SD increase in the log-scale concentration, were transformed to a 1 SD value[15]. From the pooled risk estimates (Fig 5), concentration of uNGAL was identified as an independent risk factor of all-causes of mortality among patients with CKD (pooled adjusted RR, 1.10, [95%CI, 1.03 to 1.18], p = 0.001).

### Publication bias and GRADE classification

**Fig 6:**
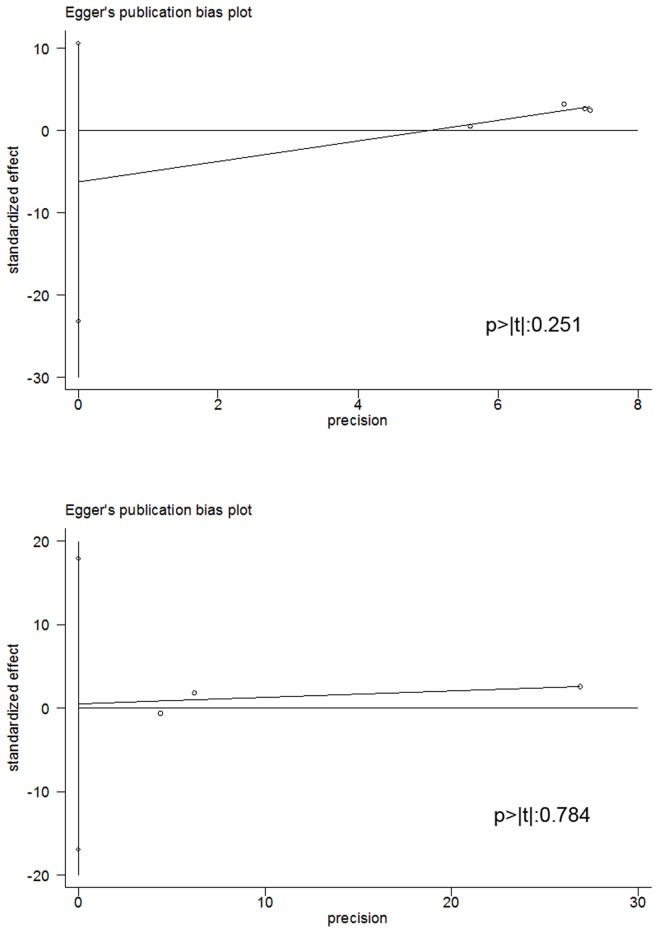
Egger’s publication bias plot. The figures show that there is no evident publication bias for the analysis of: (A) the predictive value of uNGAL for ESRD; and (B) the predictive value of uNGAL for mortality.

Egger’s funnel plot and the Egger’s linear regression test are shown in Fig 6, with no indication of significant publication bias for the analysis of the concentration of uNGAL as a predictive factor of ESRD (p>|t|: 0.251) and mortality (p>|t|: 0.784). There was also no evidence of publication bias for all other analyses (S2 Fig).

The main findings, and the level of evidence, of our systematic review are summarized in Table 2. Among our findings, only the predictive value of uNGAL for ESRD was supported by level A evidence, with the level of evidence for other findings being insufficient to recommend their utility in practice.

**Table 2:** Summary Table of Findings.

Quality assessment							No of patients	Effects		Quality
No of studies	Design	Risk of bias	Inconsistency	Indirectness	Imprecision	Association		RR	95% CI	
**CKD stage 3 (predicted with: uNGAL)**										
3	observational studies	serious	not serious	not serious	not serious	none	24748	1.06	0.96~1.18	VERY LOW
**CKD stage 3 (predicted with: uKIM-1)**										
3	observational studies	serious	not serious	not serious	not serious	weak	24748	1.13	1~1.27	VERY LOW
**ESRD (predicted with: uNGAL)**										
4	observational studies	serious	not serious	not serious	not serious	strong	19911	1.40	1.21~1.61	MEDIUM
**ESRD (predicted with: uKIM-1)**										
3	observational studies	serious	not serious	not serious	not serious	none	16402	1.13	0.96~1.33	VERY LOW
**ESRD (predicted with: uNAG)**										
3	observational studies	serious	not serious	not serious	not serious	none	16201	1.10	0.93~1.31	VERY LOW
**Mortality (predicted with: uNGAL)**										
3	observational studies	serious	serious	not serious	not serious	weak	4119	1.10	1.03~1.18	VERY LOW

Notes: No, number; RR, relative risk; 95% CI, 95% confidence interval

## Discussion

Accurate prediction of patients at high risk for adverse CKD-related outcomes poses a significant challenge to nephrologists in practice. As tubular epithelial cells (TECs) play a crucial role in the pathogenesis of CKD progression, tubular injury markers, such as NGAL, KIM-1 and NAG, are expected to be useful. However, discrepancies between several recent prospective studies have resulted in controversy regarding the potential clinical value of these markers. Therefore, in our systematic review, we sought to provide a more persuasive argument to this debate. The main findings of our systematic review and meta-analysis were as follows: (1) uNGAL was identified as an independent predictor for ESRD after full-adjustment of pooled data; (2) uNGAL was associated with the risk of mortality, from all causes, in patients with CKD; (3) uKIM-1 showed borderline significance in predicting incident CKD stage 3 independently in community-based population, whereas uNGAL and uNAG failed to show predictive value with statistical significance; and (4) considering the grade of evidence, only uNGAL, as a predictive factor of ESRD, was supported by medium level evidence, with an insufficient level of evidence to recommend all other factors for practice.

The independent association of uNGAL with ESRD and mortality likely reflects the role of NGAL in both renal function and injury. With regards to renal pathology, recent studies have described a crucial role of NGAL in the progression of CKD by inducing apoptosis and mediating mitosis[18, 19]. The net effects of KIM-1 and NAG on renal injury, however, are largely undetermined[13, 20]. Therefore their increase in urine may be a mere indicator of tubular injury that resulted from albuminuria or hyperglycemia, which fail to add considerable predictive value to the established risk factors. Another interesting phenomena is that uNGAL seems to be less useful in predicting CKD stage 3 in community-based population, which may be due to the fact that its concentration can be altered in a plethora of other diseases [21].

The strengths of our systematic review should be acknowledged. Foremost, this is the first meta-analysis evaluating the prognostic role of tubular injury markers in CKD. As well, the studies included in our meta-analysis had high methodological quality and involved large size sample. Lastly, we applied rigorous methodology to minimize effects of statistic heterogeneity on results.

Although we did not identify significant statistical heterogeneity, we do acknowledge the potential risks of bias in our analysis due to between-study differences in sample population, measurement of biomarkers, outcome definitions and statistical models. Specifically, in our analysis of the prognostic value of KIM-1 and NAG for ESRD, the study sample was comprised of patients with diabetic kidney disease (DKD) and a general community-based population which may not have been sufficiently representative of CKD. There was also a notable variation in the measurement of kidney function among studies, including: the use of GFR measured by non-radioactive iothalamate in one study[13]; and use of the eGFR calculated using the Chronic Kidney Disease Epidemiology Collaboration (CKD-EPI) equation in three studies[12, 16, 17]and using the Modification of Diet in Renal Disease (MDRD) study equation in six. It is important to note that of the three studies evaluating the incidence of CKD stage 3, none used the CKD-EPI equation whose accuracy has been shown to be superior to that of the MDRD equation in patients with early CKD stages[1]. It is also noteworthy that three different definitions of CKD stage 3 were used, either with or without restriction of minimal decline. Moreover, two studies having a shorter follow-up period, used ‘halving of the eGFR as a surrogate endpoint for the incidence of ESRD in their analysis[14, 16]. The validity of the surrogate endpoint has recently come under scrutiny. According to a recent analysis, the ‘doubling’ of the serum concentration of creatinine, which is equivalent to a 57% decline in eGFR according to the CKD-EPI equation, occurred in only 0.7% of patients with advanced CKD over a 2-year period, accounting for only 11% of ESRD events[22]. These results confirm the low efficiency of the surrogate endpoint. Moreover, different methods to measure biomarkers were adopted. For example, CLIA and ELISA were used to measure the concentration of uNGAL by different studies, respectively. However, the CLIA method had a lower sensitivity than the ELISA method, which would bring bias.

Unqualified adjustment of statistical models in individual studies may also have introduced bias. As examples, one study used in our analysis failed to adjust measures of kidney function for ESRD, with another study not adjusting their model of mortality to cardiovascular disease[13]. Furthermore, one Cox model for mortality included 43 events for 14 adjusted variables, resulting in 3.07 events per variables(EPV)[7]. Statistically, the Cox model was insufficient to ensure validity, with a minimum of 5 events per variable generally accepted as a minimum criterion for model validity.

Other limitations of our systematic review include: a limited number of articles in each analysis and inability to analyze other important adverse outcomes, such as cardiovascular disease and rapid renal decline, or other tubular injury markers, such as L-FABP, owing to the restricted number of qualified articles available. Moreover, populations were not ethnically representative in most studies, which may limit the generalizability of our results.

Despite these limitations, our work does offer a comprehensive overview of the prognostic value of tubular markers in CKD. Our analysis suggested that uNGAL could be used in practice as an independent predictor of ESRD among CKD patients. Other novel tubular injury markers include trefoil factor 3 (TFF-3), cystatin C (cysC), retinol binding protein (RBP), β2-microglobulin, and IGFBP-7[23–25]. Well-designed prospective studies are warranted to validate their prognostic role. From our analysis, we further recommend that future studies be conducted and reported using a uniform framework, with emphasis on a common study design, measurement of biomarker concentration and statistical methods. Such a standardized approach would facilitate consensus regarding the performance of prognostic biomarkers among the clinical and research communities.

## Supporting Information

S1 PRISMA Checklist PRISMA checklist. (DOC)

S1 Algorithm Calculation procedure for the transformation of risk estimates, based on another scale, to a 1 SD increase value. (DOC)

S1 Fig Unadjusted risk estimates for uNGAL concentration in predicting ESRD. (A) Pooled unadjusted risk estimates of a 1 SD increase of the log-transformed uNGAL concentration to the incidence of ESRD. (B) Pooled unadjusted risk estimates of a 1 SD increase of the log-transformed uKIM-1 concentration to the incidence of ESRD. (C) Pooled unadjusted risk estimates of a 1 SD increase of the log-transformed uNAG concentration to the incidence of ESRD.
(DOC)

S2 Fig Egger’s publication bias plot and Egger’s linear regression test. The figures show that there is no evidence of significant publication bias among studies used in the analysis of (A) uNGAL and (B) uKIM-1 for predicting CKD stage 3, and (C) uKIM-1 and (D) uNAG for predicting ESRD.
(DOCX)
