# Supplementary material for: Are Urinary Tubular Injury Markers Useful in Chronic Kidney Disease? A Systematic Review and Meta Analysis
Source: PLoS One. 2016 Dec 1;11(12):e0167334. doi: 10.1371/journal.pone.0167334 (PMC5131971; doi:10.1371/journal.pone.0167334)
Supplement: S1 Algorithm — (DOC) [file pone.0167334.s002.doc]

**SUPPLEMENTAL MATERIAL_algorithm**

Calculation procedure for the transformation of risk estimates, based on another scale, to a 1 SD increase value.

In the case of ‘doubling’ of a concentration of biomarker as a unit increase, let ‘X’ denote the concentration of the biomarker and Y=log_2_(X). Then we have:


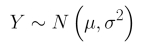


As the 25th and 75th percentile of ‘X’ are given, the corresponding percentile Y_25_ and Y_75_ limits can be calculated. From these, the value of two parameters of the distribution can be calculated:


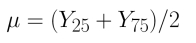


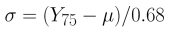
,

where 0.68 is the value of a random variable with a standard normal distribution and a cumulative probability is 0.75.

Then the risk estimates can be calculated as:


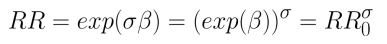


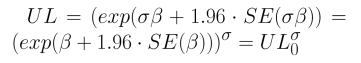


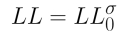
,

where β is the coefficient of Y in the original model, SE is the standard error of the mean, and with RR_0_, UL_0_ and LL_0_ denoting the relative risk, upper interval limit and lower interval limit, respectively, of the original model. The risk estimates for a 0.1 SD increase in concentration can be calculated accordingly.
