# Supplementary material for: Are Urinary Tubular Injury Markers Useful in Chronic Kidney Disease? A Systematic Review and Meta Analysis
Source: PLoS One. 2016 Dec 1;11(12):e0167334. doi: 10.1371/journal.pone.0167334 (PMC5131971; doi:10.1371/journal.pone.0167334)
Supplement: S1 Fig — (A) Pooled unadjusted risk estimates of a 1 SD increase of the log-transformed uNGAL concentration to the incidence of ESRD. (B) Pooled unadjusted risk estimates of a 1 SD increase of the log-transformed uKIM-1 concentration to the incidence of ESRD. (C) Pooled unadjusted risk estimates of a 1 SD increase of the log-transformed uNAG concentration to the incidence of ESRD. (DOC) [file pone.0167334.s003.doc]

A


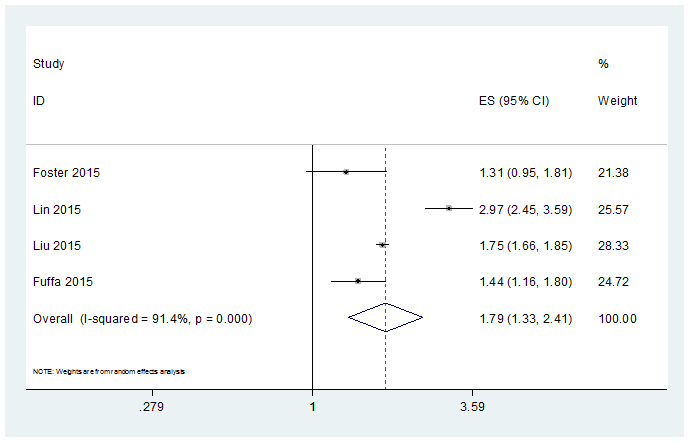


B


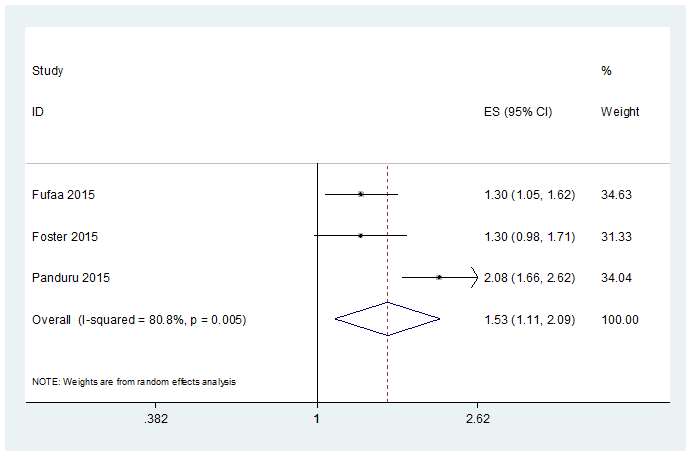


C


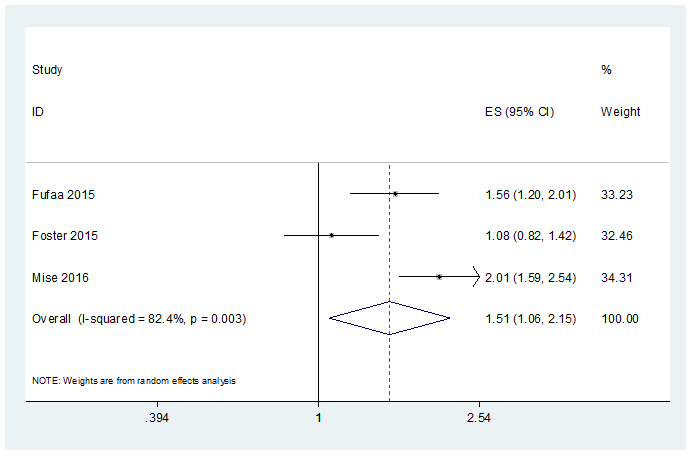


S1 Figure. Unadjusted risk estimates for uNGAL concentration in predicting ESRD. (A) Pooled unadjusted risk estimates of a 1 SD increase of the log-transformed uNGAL concentration to the incidence of ESRD. (B) Pooled unadjusted risk estimates of a 1 SD increase of the log-transformed uKIM-1 concentration to the incidence of ESRD. (C) Pooled unadjusted risk estimates of a 1 SD increase of the log-transformed uNAG concentration to the incidence of ESRD.
