# Supplementary material for: Are Urinary Tubular Injury Markers Useful in Chronic Kidney Disease? A Systematic Review and Meta Analysis
Source: PLoS One. 2016 Dec 1;11(12):e0167334. doi: 10.1371/journal.pone.0167334 (PMC5131971; doi:10.1371/journal.pone.0167334)
Supplement: S2 Fig — The figures show that there is no evidence of significant publication bias among studies used in the analysis of (A) uNGAL and (B) uKIM-1 for predicting CKD stage 3, and (C) uKIM-1 and (D) uNAG for predicting ESRD. (DOCX) [file pone.0167334.s004.docx]

A


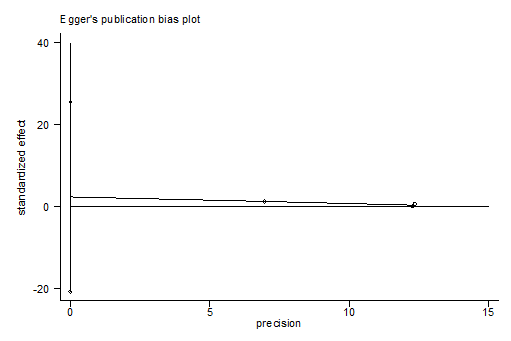


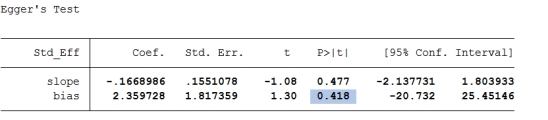


B


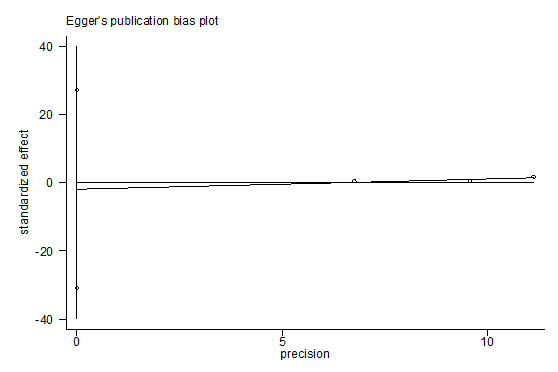


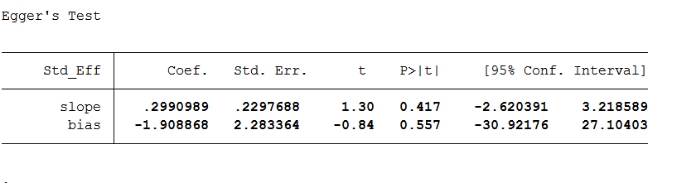


C
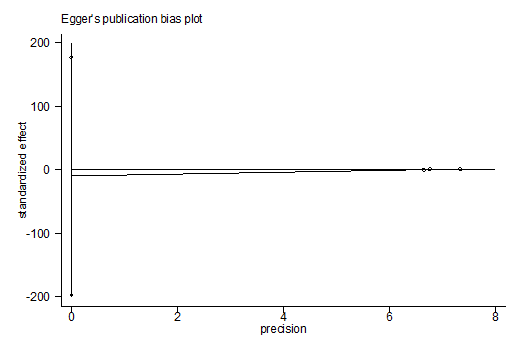


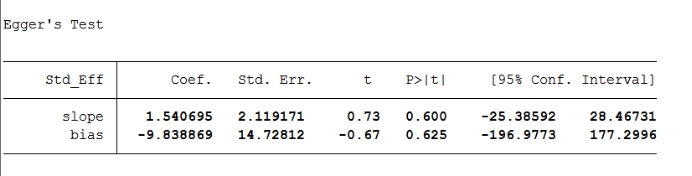


D


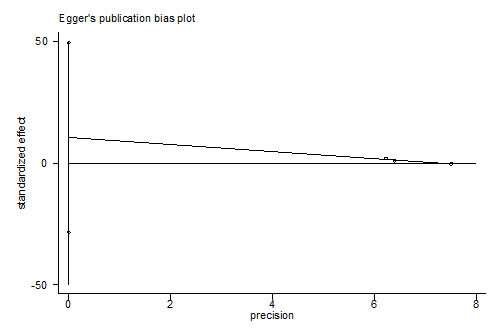


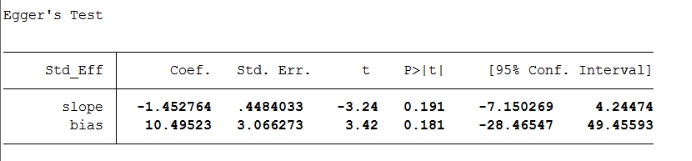


Figure S2. Egger’s publication bias plot and Egger’s linear regression test, with no evidence of significant publication bias among studies used in the analysis of (A) uNGAL and (B) uKIM-1 for predicting CKD stage 3, and (C) uKIM-1 and (D) uNAG for predicting ESRD.
